# Urban environment influences on stress, autonomic reactivity and circadian rhythm: protocol for an ambulatory study of mental health and sleep

**DOI:** 10.3389/fpubh.2024.1175109

**Published:** 2024-02-05

**Authors:** Andrea Montanari, Limin Wang, Amit Birenboim, Basile Chaix

**Affiliations:** ^1^Institut Pierre Louis d'Epidémiologie et de Santé Publique (IPLESP), Sorbonne Universités, Paris, France; ^2^Institut National de la Santé et de la Recherche Médicale (INSERM), Paris, France; ^3^Department of Geography, The Hebrew University of Jerusalem, Jerusalem, Israel

**Keywords:** environmental stress, circadian rhythm, sleep, mental health, wearable sensors

## Abstract

**Introduction:**

Converging evidence suggests that urban living is associated with an increased likelihood of developing mental health and sleep problems. Although these aspects have been investigated in separate streams of research, stress, autonomic reactivity and circadian misalignment can be hypothesized to play a prominent role in the causal pathways underlining the complex relationship between the urban environment and these two health dimensions. This study aims at quantifying the momentary impact of environmental stressors on increased autonomic reactivity and circadian rhythm, and thereby on mood and anxiety symptoms and sleep quality in the context of everyday urban living.

**Method:**

The present article reports the protocol for a feasibility study that aims at assessing the daily environmental and mobility exposures of 40 participants from the urban area of Jerusalem over 7 days. Every participant will carry a set of wearable sensors while being tracked through space and time with GPS receivers. Skin conductance and heart rate variability will be tracked to monitor participants' stress responses and autonomic reactivity, whereas electroencephalographic signal will be used for sleep quality tracking. Light exposure, actigraphy and skin temperature will be used for ambulatory circadian monitoring. Geographically explicit ecological momentary assessment (GEMA) will be used to assess participants' perception of the environment, mood and anxiety symptoms, sleep quality and vitality. For each outcome variable (sleep quality and mental health), hierarchical mixed models including random effects at the individual level will be used. In a separate analysis, to control for potential unobserved individual-level confounders, a fixed effect at the individual level will be specified for case-crossover analyses (comparing each participant to oneself).

**Conclusion:**

Recent developments in wearable sensing methods, as employed in our study or with even more advanced methods reviewed in the Discussion, make it possible to gather information on the functioning of neuro-endocrine and circadian systems in a real-world context as a way to investigate the complex interactions between environmental exposures, behavior and health. Our work aims to provide evidence on the health effects of urban stressors and circadian disruptors to inspire potential interventions, municipal policies and urban planning schemes aimed at addressing those factors.

## Introduction

### Background and rationale

By 2050 more than 70% of the world population will be living in urbanized areas (1). This fast-surging trend will significantly change the environment and expose larger parts of the global population to the health hazards that proliferate in highly urbanized areas, with serious consequences for public health (2) but also to the health opportunities in these environments. Physical factors widely distributed in the urban environment, such as nocturnal light, air and noise pollution, increased heat and more generally the lower presence of green and blue areas, have been associated by several studies with higher prevalence of mood and anxiety symptoms and with worse sleep conditions (3–8). Social science studies have focused instead on the individuals' experience and perception of social cohesion, safety and risk of violence within their neighborhood and found associations with symptoms of mental disorder and worse sleep outcomes (9–12). Moreover, these urban risk unequally distributed across neighborhoods seem to exercise a differential impact on different strata of the population, with ethnic minorities and low socioeconomic status groups experiencing the greatest effect (13–17). Disparities in mental health and sleep outcomes seem to follow a comparable pattern of geographical distribution than the different socioeconomic groups within cities across affluent and disadvantaged areas. In other words, the specific distribution of environmental stressors within the city could contribute to explain, in addition to socioeconomic stressors themselves, the mental and sleep health disparities observed between neighborhoods, and could play active role in the preservation of such inequality (18–20). Converging evidence suggests that living in disadvantaged neighborhoods or neighborhoods with environmental stressors is associated with a range of poor health outcomes, from cardiometabolic conditions to mental and sleep disorders, which might surge in comorbidity with each other reinforcing and maintaining unhealthy status and maladaptive behaviors (21–23). While cardiovascular urban risk has been inquired extensively (14, 24, 25), there is still a lot of progress to be reached in the investigation of the environmental determinants of mental and more particularly sleep disorders. These two health dimensions have often been investigated separately, thus overlooking the large synergic interactions that inevitably arise between the symptoms of these different problems (26–28). Not only mental and sleep disorders might reinforce each other in their symptomatic expression and health complications, but they also appear to share similar environmental and behavioral determinants (29, 30). One of the reasons may be that the urban environment can affect common health physiological systems shared by the two health dimensions through similar causal mechanisms.

Stress, in particular, has been hypothesized to play a prominent role in the potential causal pathways underlining the complex relationship between the urban environment and health (31). Living in urban environments, and particularly in specific neighborhoods (e.g., disadvantaged neighborhoods or neighborhoods with specific urban infrastructures), is associated with repeated exposures to environmental stress (e.g., pollution, noise, social stress etc.), which can burden over time the individual health physiological function by affecting the neuroendocrine-immune axis. Specifically, cumulative loads of stress can lead to maladaptive alterations of the hypothalamic-pituitary-adrenal axis and neuroendocrine dysregulation observed in mental and sleep disorders (32–34). In parallel, chronic stress can also lead to enhanced autonomic reactivity in the form of heightened sympathetic reactivity and withdrawal of parasympathetic activity (35). Such autonomic imbalance contributes to the process of stress sensitization that is often associated with psychopathology (e.g., mood and anxiety disorders) and sleep disruption (36, 37). In light of these intuitions, the present study will investigate the environmental determinants of stress and their consequent influence on autonomic reactivity, and through it on mental health and sleep quality.

City dwellers experience less sunlight and are exposed to a much greater extent to those environmental circadian disruptors that are overly present in urbanized areas (38). There is large evidence that those living in highly illuminated areas at night, mostly in densely populated cities, tend to experience later sleep onset, reduced melatonin secretion and are more likely to develop sleep problems (39, 40). In our contemporary age, the circadian rhythm is threatened furthermore by the ubiquitous presence of digital devices. It has been proven that exposure to the blue light emitted by digital screens (smartphones, TVs, computer screens, and tablets) induces night-time alertness and inhibits melatonin secretion (41, 42), resulting in circadian misalignment by shifting the timing of sleep (43, 44). These findings highlight the need to investigate individual light exposure both outdoor and indoor while tracking at the same time the individual usage of blue light emitting devices.

Circadian rhythm disruption (CRD) is not only associated with sleep alterations (40) but appears to be a common underlying factor across most mental health disorders. This is due to the detrimental consequences of the associated sleep loss and the dysregulation of the neurobiological systems involved in the regulation of emotions and stress that are subject to circadian control (27, 45, 46). The correlational nature of these findings stresses the need for developing a methodological and data collection framework that could track these different health dimensions and would permit to take a step forward in investigating the directionality of the relationships between sleep, circadian rhythm and mental health.

The evidence on the neighborhood-related risk provided by the scientific literature is mostly based on population studies that do not rely on a continuous monitoring of the environmental exposures across space and time. Most studies assessed personal exposures solely at the residential neighborhood level, whereas city-dwellers spend large parts of their daily time outside of their residential area (47, 48). The present project aims at overcoming such restricted focus on residential neighborhoods by adopting a continuous monitoring framework for assessing environmental exposures without misclassifying personal exposures. To do so, on the grounds of previous work (49, 50) the chosen methodology relies on Global Positioning System (GPS) receivers to geographically follow and contextualize participants' exposures over their daily mobility. GPS tracking will be adopted in combination with questionnaires delivered via smartphone to test participants' subjective perception through geographically explicit ecological momentary assessment (GEMA) (51). In addition, a set of wearable sensors will be carried by participants to achieve real-time ambulatory monitoring of psychophysiological parameters as a way to dynamically follow their responses to their daily environments. This innovative method will make it possible to analyze the spatiotemporal context and the causal effects of momentary objective exposures to selected environmental factors. In conclusion, the cutting-edge essence of the present work pertains to the high spatiotemporal resolution of its environmental exposure assessments and to the broad range of intermediate physiological processes and health outcomes included for the first time in one single study.

Understanding the intricate relationship between urban living and mental health, sleep quality, and overall wellbeing is of paramount importance in today's rapidly urbanizing world. As urban populations continue to grow, the impact of the urban environment on individuals' health cannot be underestimated. Previous research has highlighted the association between living in urban areas and the increased likelihood of experiencing mental health issues and sleep disturbances. However, to develop effective interventions and policies aimed at improving the health and wellbeing of city dwellers, we need a deeper and more comprehensive understanding of the underlying mechanisms. This study seeks to fill crucial gaps in the current body of knowledge by employing innovative ecological methods and wearable sensor technology to assess the real-world impact of environmental stressors and circadian disruptors on individuals' mental health and sleep quality. By investigating these interrelated factors at a high spatio-temporal resolution, we aim to uncover the specific environmental mechanisms that contribute to the complex web of urban health challenges. Ultimately, this research will not only enhance our understanding of urban health but also provide actionable insights for policymakers, urban planners, and healthcare professionals to develop strategies that promote healthier urban living environments and improve the quality of life for millions of city residents worldwide.

As we delve into the complex relationship between urban living, mental health, and sleep quality, our study endeavors to apply the exposome framework to explore the intricate interplay between environmental stressors, circadian disruptors, and various health outcomes. The exposome refers to the entirety of environmental exposures individuals encounter throughout their lives, including factors such as pollutants, behavior and psychosocial stressors that collectively influence health and contribute to the development of diseases over time (52). In this context, the urban exposome is conceptualized as the continuous spatiotemporal monitoring of quantitative and qualitative indicators associated with the various urban domains that shape the quality of life and the health of urban populations (53).

To incorporate the concept of exposome and encompass its multidimensional facets, this study adopts an innovative approach that goes beyond traditional population-based assessments. The sensor-based and GEMA methods leveraged in this study allow us to observe the spatiotemporal and behavioral components of urban health exposome with high-resolution data granularity and ecological validity. In conclusion, by combining the continuous monitoring of environmental exposures, individual behaviors, and physiological responses, our research strives to capture a portion of the exposome, to shed light on the intricate interplay between urban living, mental health, and sleep quality.

### Objectives

The present study was developed within the SURREAL project (Systems approach of urban environments and health) to investigate the complexity of the conundrum linking the environment to mental and sleep health with a multidisciplinary approach based on sensor and mobile methods. As detailed in Appendix 1, our primary goal is to investigate the environmental determinants of urban stress and its impact over time on autonomic reactivity and circadian misalignment, and through them on sleep quality and mental health via both between- and within-participant comparisons through a repeated measure framework of 7 days. The protocol allows monitoring the daily fluctuations of sleep and mental health outcomes to better understand the reciprocal influence that these two health dimensions may exert on each other. In addition, the timing of environmental exposures and health outcomes will be biologically contextualized by tracking participants' circadian rhythm and entrainment to light (i.e., the level of synchronization of the inner circadian rhythm to the light cycle as an environmental indicator of time). Our study combines mobile sensing methods (54, 55) for an accurate assessment of momentary changes in health outcomes in the urban environment. Our setup (see Table 1) aims to track participants during their daily urban activities and disaggregate their exposures, behavior and health outcomes over space and time while accounting for all possible confounders (55). The ultimate goal of such an approach is to go beyond the mere description of correlational associations and unveil the specific environmental mechanisms that cause momentary changes in sleep and mental health. Overall, our protocol will provide evidence on the health effects of urban stressors and circadian disruptors to inspire potential interventions, municipal policies and urban planning schemes that may be able to mitigate and contain them.

**Table 1:** Sensors and smartphone methods used in the study.

**Sensor/app name**	**Variable name (unit of analysis)**	**Sampling frequency**	**Analytical role (type of variable)**	**Dimension assessed**
BT-Q1000XT GPS receiver (Qstarz, Taiwan)	GPS position	One measure every 5 s for 7 days	Predictor	Environmental exposures
wGT3X+ (ActiGraph, USA)	Sedentary time (Min, %), light physical activity (Min, %), moderate to vigorous physical activity (Min, %), gross acceleration (G), metabolic equivalent of task (MET), steps	5 s epochs for 7 days	Confounder (continuous)	Physical activity (circadian rhythm)
Embrace plus (Empatica, Italy)	Heart rate, heart rate variability (beat per minute), electrodermal activity (microSiemens), skin temperature (C°)	5 s epochs (sampling frequency of 64 Hz) for 7 days	Mediator/effect modifier (continuous)	Sympathetic and parasympathetic nervous systems (autonomic reactivity), Temperature (circadian rhythm)
Dreem EEG (Dreem, France)	Sleep onset (h/m/s), total sleep time (min), wake after sleep onset (min), sleep efficiency (%), duration of sleep phases: N1, N2, N3, REM (min, %)	One measure every night for 7 days	Outcome (continuous)	Sleep (circadian rhythm)
HOBO data logger pendant temperature/light 64K, (Onset Computer, USA)	Light (Lux)	Sampling frequency of 250 Hz for 7 nights	Predictor (continuous)	Light entrainment (circadian rhythm)
SV104 noise dosimeter (Svantek, Poland)	Sound level (dB), frequency range (Hz)	One measure every minute for 7 days	Confounder (continuous)	Environmental determinant of sleep (circadian rhythm)
Thermo-Hygrometer PCE-HT 72 (PCE Deutschland GmbH, Meschede, GR)	Temperature (C°), humidity (%)	One measure every 5 s for 7 days	Confounder (continuous)	Environmental determinant of sleep (circadian rhythm)
Eco Emo Tracker (INSERM, FR)	Perceived sleep quality (Consensus Sleep diary), perceived vitality (36-Item Short Form Health Survey), perceived mood (CES-D scale), anxiety levels (State Trait Anxiety Inventory, Y-A form), environmental perception	Four EMA surveys each day for 7 days	Outcome (categorical)	Mental health Behavior Environmental perception
Smartphone tracker (HUJI, IS)	Smartphone usage (min, %)	One measure every 1 min for 7 days	Mediator/effect modifier (categorical)	Behavioral

## Methods

### Study design and setting

The data collection of the study presented in this protocol paper will take place between the last quarter of 2023 and the first quarter of 2024 in the metropolitan area of Jerusalem, Israel.

During such collection period, each participant will wear for 7 days several sensors that track passively multiple psychophysiological indicators. All the data collected will be timestamped and matched with GPS points to contextualize each measure with space-time coordinates. Participants will reply 4 times a day to smartphone questionnaires (in the form of GEMA) assessing participants' perception of the environment, mood and anxiety symptoms, sleep quality and vitality. Smartphone usage will be tracked passively for the entire duration of the study.

### Participants

#### Sampling and recruitment

Our study sample will include ~40 participants from the city of Jerusalem (Israel). We are aiming at sampling an adult population of 18–65 years of age without impairing health conditions. Individuals working at night (due to circadian rhythm disruption) or without a fixed home will be considered ineligible for the study. Multiple steps in the recruitment process and a detailed set of screening criteria will be used to filter out ineligible candidates and to ensure diversity within the sample.

Geofencing technology will be adopted to target participants from specific geographical areas of interest within selected administrative units. The small sample of this study cannot be considered “representative” of a large population of almost one million people such as in Jerusalem city. For this reason, quota sampling is an appropriate method because it aims at ensuring that the diversity of different population groups is well-represented in the sample. Thus, non-probability quota sampling will be used to recruit the participants and allocate them into several equal distinct strata. The principle of quota sampling is that when the desired number of people in a given category is reached, the recruiting of other participants in such category is stopped. Quota sampling in this study will be non-proportional because the distribution of participants between the categories of the sample will not necessarily correspond to the distribution in the population. The quota sample will be stratified according to population density, neighborhood average household income, age, and gender.

#### Study size

Our study will have a total sample size of 40 participants and will involve a repeated measures framework with a data collection period of 7 days. This sample size was determined based on a pragmatic approach that balanced the feasibility of data collection and the exploration of a wide range of associations with several outcomes. For each participant, the following measures are collected.

- 1 set of measures of sleep staging for a total of 7 measures over the week;
- 1 set of self-assessed measures of sleep quality (10 items) each day for a total of 7 measures (70 items);
- 1 set of self-assessed measures of mood, anxiety and vitality symptoms (18 items) each day for a total of 7 measures (126 items);
- 720 measurements of skin conductance and temperature (1-min measurements over 12 h) each day for a total of 5,040 measures;
- 720 measurements of heart rate and heart rate variability (1-min measurements over 12 h) each day for a total of 5,040 measures.

This data collection framework will provide the following total number of measurements for 40 participants:

- 280 measures of sleep staging;
- 280 measures of sleep quality (2,800 items);
- 280 measures of mood, anxiety and vitality symptoms (5,040 items);
- 201,600 measurements of skin conductance and skin temperature;
- 201,600 measurements of heart rate and heart rate variability;

We believe that the total number of measurements that will be available is sufficiently large to provide adequate statistical power to test our hypotheses based on repeated measurement models, regardless of the level of intra-individual correlation considered. That said, we are aware that our individual-level sample is not large enough to be considered representative of a source population of almost 1 million and that extreme caution will be needed in generalizing the results to the source population. Our protocol represents a feasibility study to test a complex methodological framework. Our study is mechanistically and etiologically innovative and has a high level of internal validity. If our innovative framework and exploratory analyses are successful, it will serve as a model framework for future studies with larger samples and other innovative data collection methods.

### Feasibility assessment

This protocol outlines a feasibility study aimed at unraveling the intricate relationships between environmental stressors, behavior, and health in an urban setting. Given the multifaceted nature of this research, it is vital to assess the feasibility of the data collection, participant recruitment, and logistical aspects inherent in the study design.

Data collection methods' reliability will be closely scrutinized throughout the study. Wearable sensors, GPS tracking, ecological momentary assessment tools, and other data collection instruments will undergo continuous evaluation to ensure their effectiveness in a real-world urban environment. Any technical issues arising during data collection will be documented and addressed promptly.

Participant recruitment and retention are pivotal to the study's success. The practicality of enrolling and retaining 40 participants for a 7-day study period will be thoroughly assessed. This sample size was chosen to strike a balance between research depth and resource availability, and this feasibility study will provide insights into maintaining participant engagement and compliance within a complex sensor-based study.

Maintaining data quality and integrity is a paramount concern. Protocols for data storage, processing, and analysis will be rigorously evaluated to safeguard the accuracy of the collected data. Continuous monitoring of participant compliance with the study protocol, including sensor wear and ecological momentary assessments, will be essential. Strategies will be implemented to optimize participant engagement and minimize attrition.

In summary, this feasibility assessment will permit to refine the methodology for future larger-scale studies by addressing practical challenges and logistical considerations related to data collection and participant engagement. The goal is to ensure the successful execution of the study, shedding light on how urban living impacts mental health and sleep quality. See Appendix 3 for more methodological details on the feasibility assessment.

### Ethics and data protection

The present study was approved by the Institutional Review Board (IRB) of the Hebrew University of Jerusalem. The personal data collected during the study will be processed and stored in accordance with the General Data Protection Regulation (GDPR) in force within the European Union. Only the research staff in charge of collecting the data will have temporary access to the participants' names and surnames which will be subsequently pseudo-anonymized and replaced with identifying encrypted codes for the subsequent phases of the study. In this way, the people in charge of analyses will not be able to access participants' credentials and identities. The data will be aggregated and digitally stored for 15 years in high-security servers hosted within Israel and the European Union and permanently destroyed at the end of the authorized period. Only named researchers from the two teams will be able to access the data after signing a protection and confidentiality clause. This procedure will ensure that the data will be stored in a totally private and confidential manner.

### Sensor and smartphone-based strategy—variables and measurements

**Figure 1:**
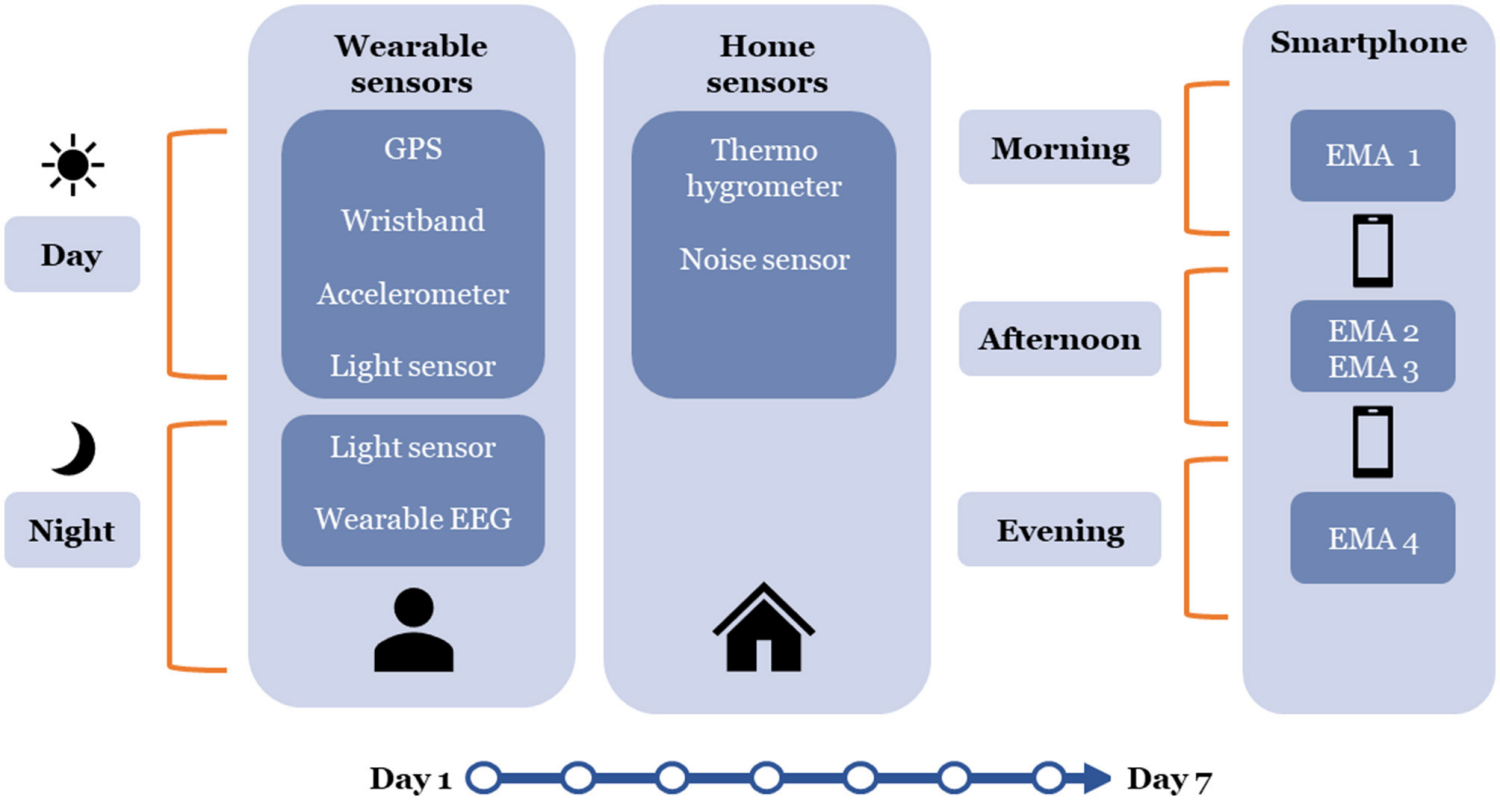
Overview of the data collection.

See Figure 1 for a synthesis of our wearable sensing strategy. For a comprehensive summary of all assessed study variables (including variable names and their unit of analysis, the sensors used and their sampling frequencies, the analytical roles and categorization of variables, and dimensions assessed) please consult Table 1.

In this study, data will be collected from a diverse array of sensors. We will acquire data at a very fine-grained time frequency, i.e., as much as needed for the construction of the most meaningful environmental exposure and physiological variables, while also taking into account constraints related to the storing of data in these sensors. Sensitivity analysis will enable us to assess the impact of different time windows of aggregation of environmental and physiological data on the meaningfulness of our variables, statistical power available, and precision of our analyses. Data will also be aggregated on the basis of hypotheses of time frames over which specific environmental variables, behaviors, and outcomes interact with each other. This rigorous approach ensures that our study leverages the full potential of sensor technology while maintaining the highest standards of scientific rigor and interpretability in our findings. For an in-depth description of our approach to modeling study variables in our analysis, please refer to Appendix 3.

#### Autonomic regulation and circadian monitoring

##### Heart rate variability, skin conductance, and temperature

Every day, participants will carry the Empatica Embrace Plus bracelet, a non-invasive user-friendly wristband developed for research and clinical studies. The internal sensors of the wristband allow real-time detection of heart rate and heart rate variability (HRV; estimated from blood volume and interval between beats, respectively), electrodermal activity and peripheral body temperature (or skin temperature). The device also includes a three-axis accelerometer to track movement and assess users' activity.

The combination provided by the Embrace Plus of HRV, which reflects the joint influence of the sympathetic and the parasympathetic nervous systems on the heart (56), and of electrodermal activity, which indicates sympathetic arousal (57), allows to assess in real-time the dynamic tonal balance of the two branches of the autonomic nervous systems in response to environmental stressors (58, 59). These two measures together could provide an indirect measure of participant's emotional functioning, autonomic regulation and reactivity to stress (60, 61) and ultimately of their physiological adaptability to the environment (62, 63).

##### Circadian phase assessment by ambulatory monitoring

Each day participants will carry a light-intensity data logger (HOBO Data Logger) that continuously tracks ambient light exposure. During the day participants wear the sensor on the neck collar of their clothes through a safety pin to get as close as possible to the eye level. In the context of this study, light exposure is more accurately measured at the eye level compared to other parts of the body (e.g., the commonly used wrist) as it reflects more precisely the transmission of light from the eye retina to the suprachiasmatic nucleus which is the central circadian peacemaker (64–66). Lux intensity (measured in lumen) and time of exposure will be used to classify participants' state of entrainment to sunlight. Following previous protocols, to compensate for the lack of light spectrum detection with the chosen device, measurements over 1000 Lux will be considered as sun exposure whereas lower lux counts will be classified as artificial light (67). Following previous protocols, skin temperature and accelerometer, which are both input and output circadian signals, and light exposure, which is an input signal, will be used for continuous ambulatory circadian monitoring of participants (68, 69). In the absence of hormonal samples to directly measure the effects of circadian alignment on the phases and amplitudes of the main circadian hormones (such as melatonin or cortisol), ambulatory circadian monitoring and sleep time patterns (onset and mid-sleep) are used instead as the main proxies for circadian entrainment (i.e., the synchronization of the internal biological clock to external time cues, such as the natural dark-light cycle).

#### Sleep assessment

##### Wearable electroencephalography (EEG)

Each night, participants' sleep onset, sleep efficiency, total sleep time, and duration of sleep stages [N1, N2, N3, rapid eye movement (REM)], will be assessed through the wearable user-friendly EEG Dreem Headband. Sleep staging performance of this device has been validated against the gold standard polysomnography sleep experts' manual scoring (70). The headband offers high sleep staging accuracy in a non-intrusive user-friendly device for ecological self-assessment in the domestic environment. Thus, it represents an improvement in sleep assessment compared to the widely used actigraphy-oximetry combination which struggles to distinguish between motionless, wakefulness, and other sleep stages (70–72). In this study, while sleep quality is one of our main outcomes, every aspect related to the timing of sleep (beginning, midpoint, and end times of sleep) pertains to the circadian assessment, which is one of our explanatory processes of interest.

##### The sleep context: ambient noise, temperature, humidity, and light

Between-household differences have been documented for sleep outcomes. One reason is that the inner environment of the houses can strongly affect the ambient temperature, air quality, noise, ventilation and light conditions of the sleep setting (18, 73). To account for these environmental factors, each night the SV104A dosimeter mounted on a tripod will be used to monitor the ambient noise of the participants' bedrooms while a thermo-hygrometer is used to register the ambient temperature and the humidity levels of their sleeping environment. This set of sensors, combined with the light sensor, will allow us to account for important environmental factors that may affect sleep hygiene and quality.

#### Geographically explicit ecological momentary assessment

##### Location and physical activity

Participants will carry a BT-Q1000XT GPS receiver and a wGT3X+ tri-axial accelerometer on an inelastic waist belt over the 7 days of tracking. These two sensors will allow tracking participants through space and time while controlling for physical activity intensity.

The accelerometer will be positioned on the right hip of each participant to capture precise data related to physical activity and movement patterns throughout the study duration. Data collection using these two sensors occurs continuously over 7 days, encompassing all waking hours. To ensure the validity of accelerometer data and distinguish between periods of activity and non-activity, we will apply the Choi algorithm (74). The minimum number of valid days of accelerometer data required for the analysis will be defined as having at least 4 days with a minimum of 10 h of wear-time per day. These criteria are established to guarantee an adequate representation of participants' physical activity patterns while accommodating potential variations in daily routines. The accelerometer data collected will be utilized to calculate several physical activity outcomes of interest, including metabolic expenditure, time spent in moderate-to-vigorous physical activity, light physical activity, and sedentary time. These outcomes will be determined using established criteria, including the Freedson 1998 cut-points and the Crouter 2R regression equation (75–77).

##### Smartphone survey

We developed a state-of-the-art smartphone application called Eco Emo Tracker to collect momentary data on mental wellbeing and on several behavioral confounders. On each day of tracking, participants will be subjected to four electronic surveys. Each questionnaire will be triggered at random times within predefined temporal windows. A strategy for future research would be to trigger such questionnaires according to participants' approximate biorhythm [as assessed by the Munich Chronotype Questionnaire (MCTQ)].

° A first morning questionnaire will assess participants' perceived quality of sleep together with alcohol, caffeine, and medication consumption (using items from the Consensus Sleep diary).
° Two questionnaires, the first one at midday and the second one after sunset, will assess participants' perceived vitality, mood and anxiety levels (through items from the CES-D scale, the Y-A form of the State-Trait Anxiety Inventory and of the Vitality subscale of the 36-Item Short Form Health Survey). Environmental perception will also be assessed.
° A final questionnaire before bedtime will assess participants' perceived vitality, mood and anxiety levels.

Each survey will be completed within the Eco Emo Tracker smartphone application. The application permits monitoring participants' compliance with the questionnaires in real-time, enabling research assistants to intervene to encourage participation. Eco Emo tracker will also be used to administer standard web questionnaires at the inclusion, as discussed in Appendix 2.

##### Smartphone usage

A second application developed by the Hebrew University of Jerusalem will track the overall time spent using smartphone applications and the time spent using each singular family of applications to monitor participants' smartphone usage for the whole data collection period.

### Bias

In conducting our study, we recognize and address several potential biases and challenges that could impact both participants and the outcomes.

Firstly, the relatively brief 7-day monitoring period, coupled with a potential Hawthorne effect (characterized by behavioral reactivity to the protocol), and substantial procedural demands, could potentially limit our ability to comprehensively capture unaltered daily life behavior. To mitigate these concerns, we will continuously monitor data collection instruments to promptly address any technical issues and employ strategies such as reminders to optimize participants' engagement and minimize attrition. Recognizing the importance of participants' cooperation in this demanding study, we will provide substantial motivational support. Leveraging our prior experience with sensor-based data collection, we will offer robust assistance, including check-in calls during data collection to ensure both proper tool functioning and adherence to instructions. Furthermore, we will establish a dedicated support hotline, available around the clock, including evenings and weekends, to promptly address any participant's inquiries. This comprehensive support structure not only enhances the overall study experience but also serves as an effective means to overcome potential challenges tied to data collection and participants' commitment.

Secondly, our statistical models will account for individual-level confounders and temporal autocorrelation, employing robust statistical techniques and sensitivity analyses to test the robustness of findings. These models will also take into account potential time-varying confounders. For example, the analysis of relationships between objective urban stressors and mood and anxiety is susceptible to reverse causality bias, as individuals may report heightened negative environmental stressors due to their previous mental health symptoms, hindering the assessment of causal relationships. To mitigate this bias, we plan to incorporate a variable that considers prior momentary mental health state to account for potential reverse causality effects in our analyses. By implementing these strategies and addressing potential biases and challenges proactively, we aim to enhance the validity and reliability of our study's findings.

### Statistical methods and expected results

As detailed in Appendix 4, the statistical analyses of our outcome variables will follow a repeated measure framework tailored to the specific timescale of the outcome of interest. For each outcome variable (sleep quality and mental health), mixed hierarchical models including random effects at the individual level will be used. Subsequently, to control for potentially unobserved individual-level confounders, a fixed effect at the individual level will be specified for case-crossover analyses (comparing each participant to oneself). In addition, to account for systemic inter-dependencies in the data, the models will account for the endogenous and exogenous temporal autocorrelation typical of psychological and physiological measures and, when applicable, for spatial autocorrelation as well (47, 78).

**Figure 2:**
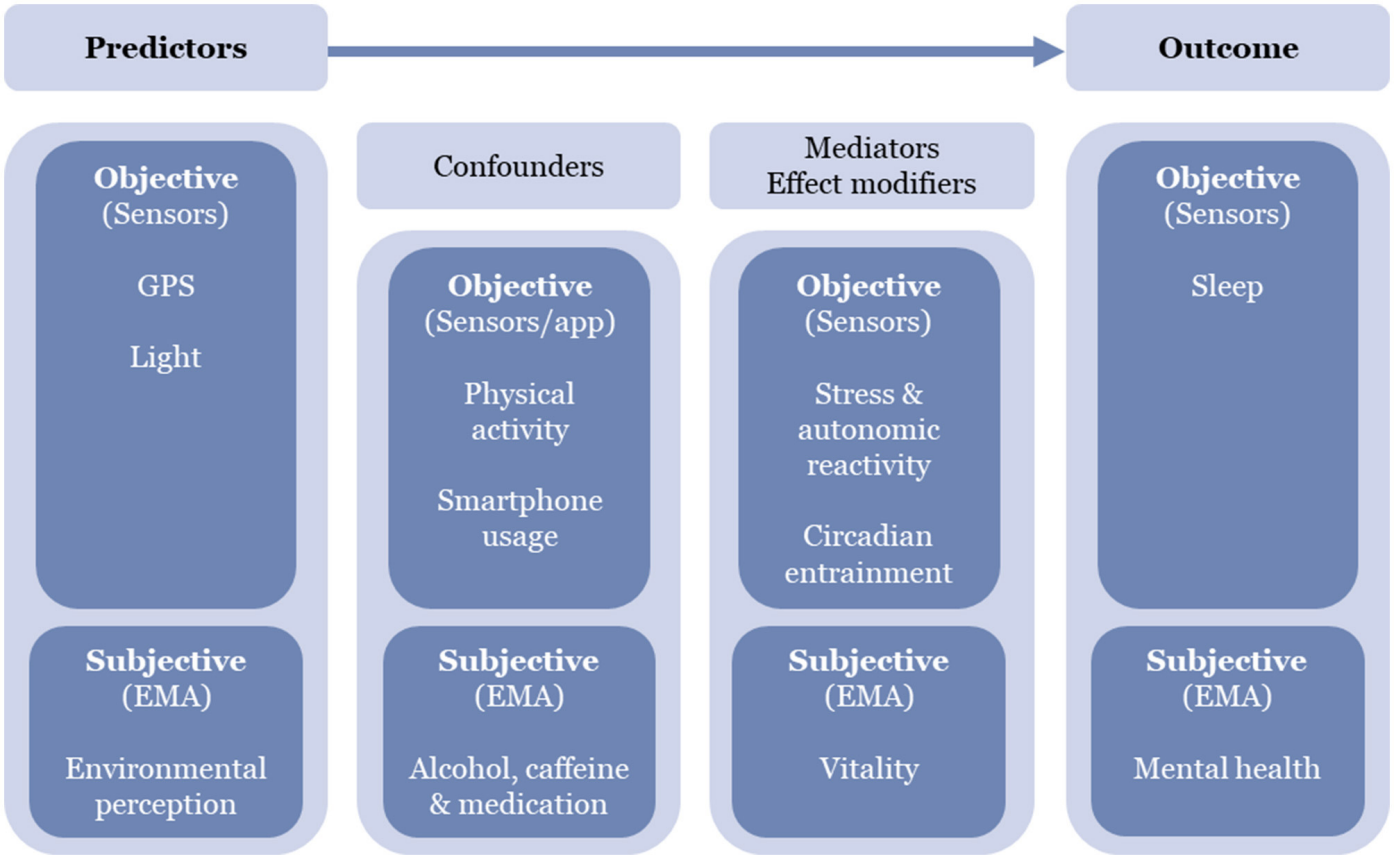
Conceptual analytical model of the study.

Firstly, separately for each outcome, we will test for associations with environmental exposures and examine the mediating role of stress and circadian entrainment in this relationship. Secondly, we will investigate the reciprocal effect that sleep and mental health may exert on each other over time by integrating in the model for each outcome the other variable. In all cases, we will incorporate into the models selected behavioral confounding and modifying factors (see Figure 2 for a detailed conceptual framework).

The application of mixed hierarchical models in the study will allow for a nuanced examination of between-individual variations in the associations between environmental exposures, behaviors, and health outcomes such as sleep quality and mental health. Moreover, exploring reciprocal effects between sleep and mental health will provide insights into their dynamic relationship over time, offering valuable insights into potential reinforcing feedback loops between these two health dimensions. Our analysis may identify specific environmental predictors, elucidate mediation or moderation pathways, and reveal temporal and spatial autocorrelation patterns. This analytical depth will contribute to a detailed understanding of the intricate interplay among our main variables of interest.

## Discussion

### Summary of strengths and limitations of the study

The present study is the first study that aims to investigate the dynamic mutual influences over time within the triad of sleep, mental health, and stress, by also monitoring the autonomic reactivity and circadian rhythm of the participants. The main strength of this approach consists in its multi-exposure and overall digital monitoring approach that comprehensively assesses through space and time individual exposures, confounding factors and health outcomes with the aid of sensors-based and GEMA methods. Exposures will be determined over day and night in the outdoor space as well as in the home environment to account for complementary types of influences. Lastly, outcomes will be assessed both objectively and subjectively to detect biases in participants' perceptions.

The current protocol presents some limitations as well. Firstly, the study entails a small sample of participants, which may limit the external validity of the results. Secondly, the short monitoring period of 7 days, combined with a potential Hawthorne effect (i.e., behavioral reactivity to the protocol) and the high burden of the procedures, might not permit to capture unbiased daily life behavior. Finally, a lack of any follow-up in the data collection will not allow us to track medium- and long-term effects of exposures.

### Future sensor-based strategies for passive ambulatory tracking

#### Innovations in and implications of autonomic tracking

Recent advances in sensor-based methods do not only provide more information on the functioning of neuro-endocrine and circadian systems in a real-world context but may also lay the foundation for a framework to investigate the embodiment of place effects and understand the relationship between individual health and the subjective and objective environment (79, 80).

Indeed, sensor-based methods allow for the evaluation of *in-situ* changes in autonomic states during spatial navigations in the environment. These states inform us about individual's “neuroception” (63), that is, the neuronal assessment of safety or danger in the environment. Thus, they could allow us to detect environmental cues that might precede anxiety or stress symptoms in the individual and, more generally, provide insight into the mind-body-environment triptych unity.

An example of innovation in autonomic regulation monitoring is the passive pupil tracking automated by the smartphone application developed by Tseng et al. (81). Such a prototype allows one to infer the influence of the two branches of the autonomic nervous system over the pupil size and thus it can be used to ecologically assess the alertness and the autonomic reactivity of individuals in response to environmental exposures (e.g., light) or behavioral factors (e.g., smartphone usage) during different circadian phases (44, 82).

#### Advancement in ambulatory circadian monitoring

Another physiological element to be leveraged in sensor-based research is human sweat. Sweat does not only allow to track electrodermal activity (83, 84) for the assessment of sympathetic arousal, it also contains several biomarkers (e.g., protein metabolites, neuropeptides, electrolytes, and cytokines) usable for non-invasive and continuous monitoring of physiological functioning and detection of unhealthy states (85, 86). For instance, the adrenal steroid cortisol produced by the hypothalamic-pituitary-adrenal axis provides information on daily stress levels and can also be used to assess how functional is the circadian clock at the endocrine system level through its cyclic diurnal rhythm (87). This hormone can be sensed through electrochemical detention by low-powered affordable wearables using devices such as the SLOCK chronobiology tracker (87) or the CATCH and Cortiwatch cortisol tracker (88, 89). These experimental sensors, by integrating assessment of hormonal biomarkers into wearable devices, provide new non-invasive tools for ambulatory stress and circadian monitoring and thus open a new area in the sensor-based research field.

**Figure 3:**
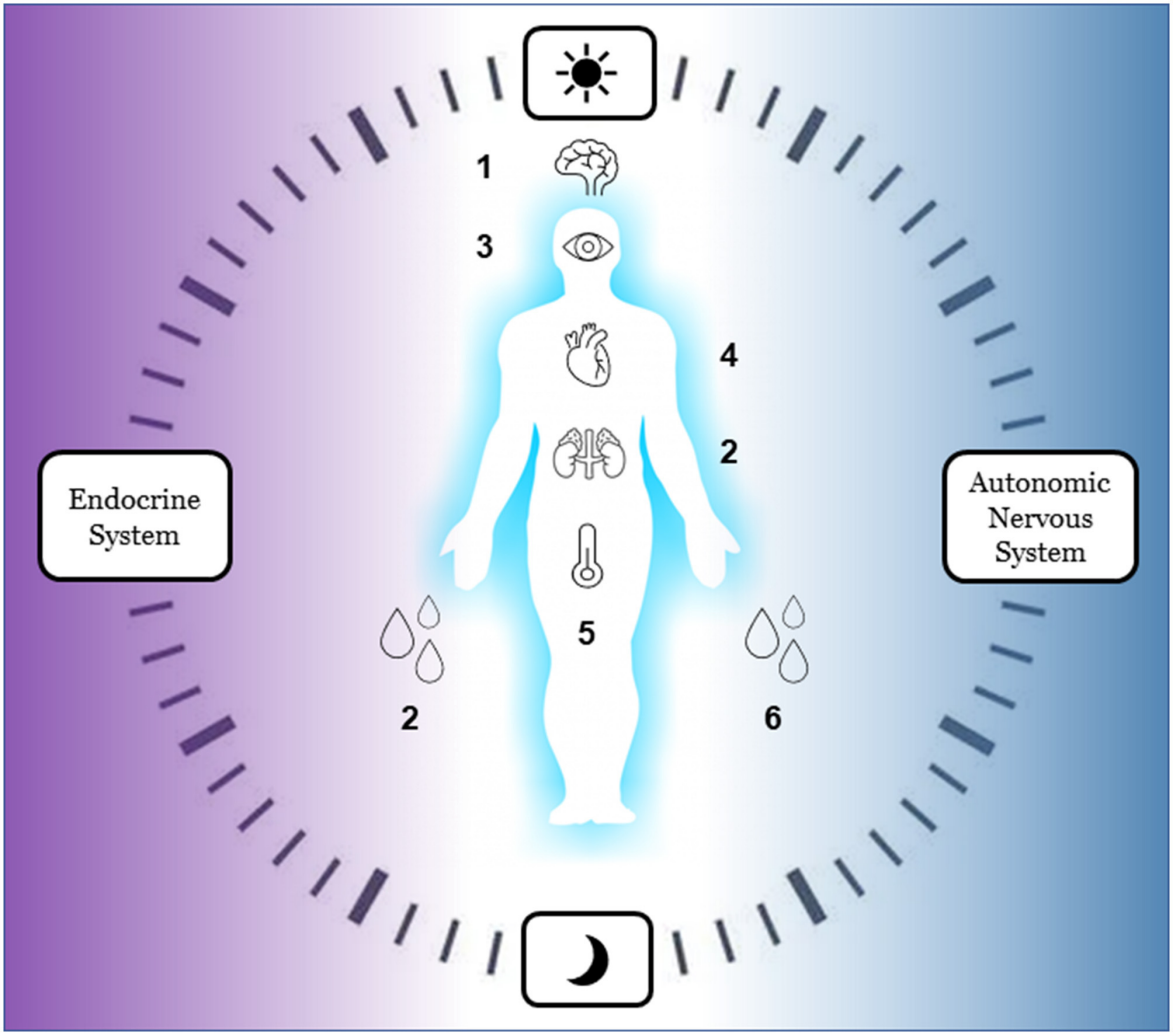
Innovative bio-signals for ecological ambulatory assessment of autonomic reactivity and circadian rhythm (to be used in future studies). Endocrine system: 1 melatonin, 2 cortisol. Autonomic nervous System: 3 pupil size, 4 heart rate variability, 5 body temperature, 6 skin conductance.

Another innovative way to integrate hormones into sensor-based research is to use melatonin samples for circadian monitoring. Dim light melatonin onset (DLMO) is a validated biomarker of circadian phase and thus it is the gold standard for the assessment of circadian phase advancement or delays in entrained individuals (90). Taking melatonin samples would complement light sensing as it allows one to connect environmental exposures to biomarkers of circadian alignment and disruption (91). The cutting-edge research in this field integrates melatonin samples with multisensor ambulatory circadian monitoring and leverages machine learning models to predict the impact of light exposure on melatonin secretion (92). Similarly, machine learning models can also be used to detect circadian rhythms of sleep, motor, and autonomic functioning from multisensor circadian monitoring (93) to predict sleep and circadian disorders (69). Although these novels methods are under development, they have yet to be integrated in protocols investigating the effects of the built, socio-environmental, and home environments on mental and sleep health (see Figure 3 for a summary of new bio-signals to be considered in future studies).

## Data availability statement

The original contributions presented in the study are included in the article/Supplementary material, further inquiries can be directed to the corresponding author.

## Author contributions

AM and LW defined the hypothesis of the study under the supervision of BC and AB. AM designed and wrote the operational protocol. All authors revised the manuscript for intellectual content. All authors contributed to the article and approved the submitted version.
