# Supplementary material for: Urban environment influences on stress, autonomic reactivity and circadian rhythm: protocol for an ambulatory study of mental health and sleep
Source: Front Public Health. 2024 Feb 5;12:1175109. doi: 10.3389/fpubh.2024.1175109 (PMC10875008; doi:10.3389/fpubh.2024.1175109)
Supplement: Supplementary file 1 [file Data_Sheet_1.docx]

# Urban environment influences on stress, autonomic reactivity and circadian rhythm: protocol for an ambulatory study of mental health and sleep

## Appendix 1

### Detailed objectives

The present sensor-based study aims to investigate the complex relationship between environmental stress exposures and selected health outcomes through an interdisciplinary approach based on Epidemiology, Geographic Information Science and Clinical Psychophysiology among others. Such protocol will allow us to provide compelling evidence on the health effects of numerous urban stressors and circadian disruptors and provide the basis to create implementational knowledge aimed at addressing those factors. Accordingly, first, we aim to inform governmental and public health agents on the health hazard posed by such threats as a mean to inspire health interventions, municipal policies and urban planning that may be able to prevent and contain them. Secondarily, we would like to individuate at-risk populations and create awareness of the health disparities experienced by those living in more adverse urban areas. Finally, we will detect and describe all those maladaptive behaviours that might play a predominant role in the maintenance of unhealthy status. Ultimately, we aim at contributing to develop health guidelines to acquire greater control on the personal environment and to counterbalance unhealthy behaviours that are promoted in urban centres.

Regarding our objectives, firstly, we aim to assess the short- and medium-term impact of selected environmental factors on cardiovascular parameters (heart rate and heart rate variability) and electrodermal activity (skin conductance response and skin conductance level) as a way to investigate the dynamic modulation of the participants’ autonomic nervous system in response to stress. The goal is to compare the effects of the social and physical environment on our participants’ health during their day-to-day mobility and environmental exposures, as a mean to investigate the critical timing and the source-specific impact of environmental stress. We also plan to address the differences in environmental stress exposures between different neighbourhoods and socioeconomics groups. Secondly, we will analyse the influence of the environment on the subjective measures of perceived stress, anxiety and mood levels through a geographically explicit ecological momentary assessment methodology (GEMA), as a way to localise and contextualise our participants’ responses. We aim to compare these subjective measures with the objective indicators of stress to study their reciprocal influence and divergence in real-time. Thirdly, we will investigate the impact of environmental stress on sleep quality. For each night of observation, we will track and assess our participants’ sleep staging, quality and duration through the electroencephalographic signal and morning electronic surveys. Subsequently, we will study the daily cumulative effects of the objective and subjective measures of stress on the selected sleep outcomes. Fourthly, we will investigate the complex relationship between sleep and mental health symptoms, with a focus on the cumulative effects recorded day after day. In addition, we aim to identify the major environmental circadian disruptors. We will assess the impact of selected environmental factors (e.g., light exposure) and behavioural factors (e.g., accelerometery) during participants’ days and nights on several circadian outcomes (e.g., sleep outcomes,). We will assess self-perceived vitality as a subjective measure of our participants’ circadian rhythm. For this purpose, we use an ecological momentary assessment methodology to assess daily fluctuations in vitality in relation to the individual’s normal biorhythm and chronotype. Lastly, we will examine extensively our participants’ usage of smartphones. We aim to identify the actual environmental factors that trigger smartphone use and to assess the resulting effects on participants’ health.

## Appendix 2

### Standard computer assisted questionnaires

Before the beginning of the data collection, under the supervision of a research assistant, the participants will fill a questionnaire specifically developed for the study covering the following topics:

- health status;
- health-related behaviours;
- socioeconomic status;
- transport habits;
- perception of the residential neighbourhood and city;
- characteristics of the domestic sleeping environment.

Before the data collection the participants will fill a battery of standardized questionnaires investigating the following domains:

- quality of life;
- perceived stress;
- mood and anxiety states and traits;
- chronotype;
- sleep quality;
- social media engagement and addiction.

These questionnaires will be used to contextualise participants’ objective data with their subjective perceptions on the dimensions inquired. Chronotypes will be considered when evaluating participant circadian rhythm.

## Appendix 3

### Feasibility assessment

#### Assessing the reliability of sensor and GEMA measures:

- We plan to calculate the percentage of missing values for each measure (regardless of whether it was obtained through sensor-based or GEMA methods), with a threshold for considering a measure as not easily feasible if the percentage of missing values exceeds 20%. Data losses greater than 20% might compromise the validity of the study, reduce statistical power and undermine the accuracy of data imputation (1–3).
- Statistical analyses will include descriptive statistics and imputation methods if necessary, aiming to ensure the reliability and completeness of collected data.

#### Practicality of enrolling and retaining participants:

- Enrolment and retention will be assessed quantitatively by calculating the percentage of participants who complete the entire study.
- A qualitative assessment will be conducted with each participant at the end of their data collection period by our research assistants to gather feedback on the burden perceived during the data collection and main causes of perceived burden.

These refinements aim to provide a comprehensive understanding of the feasibility of the study, combining both quantitative and qualitative assessments. We believe this detailed plan will contribute to a robust evaluation of our study's practical aspects.

## Appendix 4

### Statistical analyses and expected results

For the first objective, we will model the associations between the daily environmental exposures (environmental perception assessed with ecological momentary assessment and light exposures) and the psychophysiological measures of heart rate variability and electrodermal activity as the outcomes. For the second objective, we will model the associations of the daily environmental exposures and the psychophysiological measures (heart rate variability and skin conductance) with mood and anxiety (assessed with ecological momentary assessment) as the outcomes. For the third objective, we will model the relationships between, on the one hand, the daily environmental exposures, the psychophysiological measures, and light exposure levels and on the other hand the sleep measures as the outcomes. Finally, we will also take into account in this model the associations between nocturnal noise, light, temperature and humidity levels and sleep measures as the outcomes. This will allow us to contrast and compare the effects of daily and nightly exposures on sleep. For the fourth objective, we will explore two different models. The first one will use daily mental health scores as predictors and sleep measures as outcomes. The second one will test the inverse relationship using, instead, mental health variables as the outcomes. The goal of these models is to investigate the reciprocal influence that these two factors might entertain with each other over time. The model created in objective 1 will be pivotal to distinguish between direct and indirect effects of environmental exposures on mental health and sleep outcomes in objectives 2-4. A causal mediation framework will be adopted to investigate heart rate variability and skin conductance as momentary mediators between the environment and the other health outcomes. Additionally, interaction terms will be introduced in the models to test the two parameters of autonomic reactivity as effect modifiers. For the fifth objective, we will expand the previous sleep models to account for each participant’s circadian rhythm. The model will be adjusted for temporal effects as a mean to contextualise each exposure within biologically significant time windows. The period of the day (morning, afternoon, evening times, which will be defined according to the seasonality) will be used to qualify each environmental or behavioural effect of interest in relation to the individuals’ biorhythm. For the last objective, the relationship between smartphone usage and different health outcomes will be investigated. Firstly, we will model the associations between smartphone usage and mental health scores and sleep quality as the outcomes. Secondly, we will estimate an inverse model with smartphone usage as the outcome. Finally, we will test smartphone usage as a mediator or effect modifier in the relationship between the environmental exposure and the sleep and mental health outcomes

In all cases, we will integrate into the models selected behavioural confounding and modifying factors such as physical activity, alcohol, caffeine and medicine consumption, and smartphone usage. Different statistical methods (e.g. quadratic or cubic terms, piecewise regression analyses, or smoothing terms), will be adopted to model the effects of the environmental factors of interest. When relevant, multi-exposure models will be used to estimate the independent effects of concomitant environmental factors on the outcome of interest. For each outcome, a sensitivity analysis will be also conducted to estimate the appropriate temporal window to consider when assessing the exposure of interest. Individual and sociodemographic characteristics of the sample will be considered in our models. For instance, age differences will be adjusted for to account for age-related biological variation in the sleep architecture and circadian rhythm.

## Bibliography

1. Little RJA, Rubin DB. Statistical Analysis with Missing Data. John Wiley & Sons; 2019. 462 p.

2. Schulz KF, Grimes DA. Sample size slippages in randomised trials: exclusions and the lost and wayward. The Lancet. 2002 Mar;359(9308):781–5.

3. Kang H. The prevention and handling of the missing data. Korean J Anesthesiol. 2013 May 24;64(5):402–6.
